# Autoantibody test for type 1 diabetes in children: are there reasons to implement a screening program in the general population? A statement endorsed by the Italian Society for Paediatric Endocrinology and Diabetes (SIEDP-ISPED) and the Italian Society of Paediatrics (SIP)

**DOI:** 10.1186/s13052-023-01438-3

**Published:** 2023-07-19

**Authors:** Valentino Cherubini, Francesco Chiarelli

**Affiliations:** 1 Department of Women’s and Children’s Health, Salesi Hospital, Ancona, Italy; 2 Department of Paediatrics, University of Chieti, Via dei Vestini, 5, I-66100 Chieti, Italy

**Keywords:** Type 1 diabetes, Screening, Prediction, Prevention

## Abstract

In recent years screening of type 1 diabetes (T1D) in both at risk children and general population has been widely discussed with the aim of increasing awareness of the importance to early detect (and possibly treat) at-risk children in early stages of the chronic autoimmune progression to T1D.

In fact, it is well known that first-degree relatives have the highest risk of T1D, but up to 90% of children who develop T1D do not have a family history and belong to the general population.

The reasons for screening children well before the clinical onset of T1D include prevention of DKA (still up to 60% children are diagnosed with DKA) and related morbidities and mortality, reducing the need for hospitalisation, time to provide emotional support and education to ensure a smooth transition to insulin treatment, and opportunities for new treatments to prevent or delay progression.

There are pros and cons of screening of T1D in children, but recent evidences suggest that it is now time to consider possible a screening for T1D in children.

Recently, the European Society for Paediatric Endocrinology (ESPE) has endorsed a Position Statement, discussing the pros and cons of screening for T1D in the general population; ESPE supports national Societies for Paediatric Endocrinology to spread information on this important issue in various countries throughout Europe; the Italian Society for Paediatric Endocrinology and Diabetes (SIEDP-ISPED) and the Italian Society of Paediatrics (SIP) endorse this document with the specific aim of increasing awareness on screening for paediatric T1D in the general population.

**Supplementary Information:**

The online version contains supplementary material available at 10.1186/s13052-023-01438-3.

## Main text

Type 1 diabetes mellitus (T1D) is one of the most frequent chronic diseases in children; it is due to an autoimmune destruction of the insulin-producing β-cells in the islets of Langerhans within the pancreas [1]. Patients with T1D lose blood glucose control, which can result in both acute conditions (ketoacidosis and severe hypoglycemia) and chronic complications (retinopathy, nephropathy, neuropathy, and cardiovascular diseases). The pathogenesis of the T1D involves environmental factors (e.g. enteroviral infection) and polygenic predisposition.

The incidence of T1D has increased dramatically over the last five decades, especially in children younger than five years [2]. Those under the age of 18 years are most often affected, but an equal number of adults over 18 are thought to develop the disease. Currently there is no cure to T1D, therefore the patients are destined for a lifelong insulin treatment and, in most cases, the development of disease-related complications. Additionally, T1D has a huge economic burden on the patients, their families and the health systems globally [3]. Numerous studies have shown that new technologies improve glycaemic control and long-term outcomes in children and adolescents with type 1 diabetes. Furthermore, these devices have improved quality of life and have patient satisfaction [4].

Here we discuss the main unmet needs in type 1 diabetes and the opportunity presented by population screening.

## Unmet needs

New drugs and technologies promise improvements in the care of patients with type 1 diabetes; however, some major unmet needs remain on the table, including the high frequency of DKA, the continued threat of hypoglycemia, the day-to-day burden of diabetes management, and failure to achieve optimal glycaemic control.

## Diabetic ketoacidosis at clinical onset of diabetes

A recent international study showed that the frequency of DKA at diabetes diagnosis was between 20.7% and 48.7% in the years 2006–2019 with a huge increase during the COVID-19 pandemic, exceeding 55% of cases [5]. DKA is a clinical emergency associated with serious complications including cerebral edema, increased mortality rates, prolonged hospital stays, excessive costs, and poor long-term metabolic control [6] [7] [8]. A single episode of moderate/severe DKA in young children at diagnosis is sufficient to cause cognitive impairment and impaired brain growth [9]. The presence of DKA suggests delayed or unrecognized symptoms by parents or caregivers. DKA awareness campaigns are effective in reducing the frequency of DKA at the clinical onset of type 1 diabetes in children and adolescents [10]. However, large-scale implementation of prevention campaigns requires considerable effort and their diffusion is still limited today. Very interesting results on the reduction of DKA at the diagnosis of diabetes have been reported by the screening program of the general population using anti-beta-cell autoantibodies [11].

## Increased hospitalization

Hospitalization rates in children with diabetes are at least three times higher than in the general paediatric population, regardless of the presence of diabetic ketoacidosis at diabetes diagnosis, which is known to increase the likelihood of hospitalization [12]. Additionally, diabetic ketoacidosis can lead to hospitalizations after a diagnosis of diabetes. Indeed, in the first 12 months after a diabetes diagnosis, more than 1 in 20 children require rehospitalization for ketoacidosis [13].

## Burden of type 1 diabetes

Diabetes management is challenging and often overwhelming for young people with type 1 diabetes and their caregivers. Young people with diabetes appear to have a greater incidence of depression, anxiety, psychological distress and eating disorders compared to their healthy peers [14]. Optimal treatment requires a young person with T1D and his or her family to monitor dietary intake, count carbohydrates, monitor trends in daily glucose values with a sensor or capillaries multiple times a day, deliver insulin multiple times a day with a pen or a pump [15]. Advanced technologies and treatments can also add burdens, which may cause young people to stop wearing them. Such treatment burdens have the potential to significantly impact quality of life. Furthermore, lack of diabetes control and adequate insulin therapy often promotes diabetes-related family conflict, poor academic performance, and/or increased interpersonal conflict [16].

## Suboptimal glycemic control

Advances in insulin therapy, including the development of next-generation insulin analogues, targeted delivery approaches, continuous glucose monitoring (CGM), and automated insulin delivery systems have contributed to improvements in glucose control. However, for most people with type 1 diabetes, long-term glycaemic control remains suboptimal [17].

## Hypoglycaemia and fear of hypoglycemia

People with type 1 diabetes face and manage the acute and life-threatening threat of hypoglycemia on a daily basis [18]. A few months after disease onset, counterregulatory mechanisms, including a physiological decrease in insulin level and glucagon secretion, are lost [19]. Also, over time, people with diabetes may experience a decrease or complete disappearance of hypoglycaemia symptoms. Preventing hypoglycemia is difficult and requires overcoming barriers such as emotional (e.g. fear of gaining weight), educational (e.g. choice of treatment), action planning (rescue treatment at hand), and social factors (fear of attracting unwanted attention). While new drugs and technology available today allow for the reduction of hypoglycaemia, there remains a strong need for treatments that lower blood sugar to the desired target without causing hypoglycemia or weight gain. On the other hand, fear of hypoglycaemia remains one of the main factors limiting the achievement of optimal glycaemic control.

## Screening of type 1 diabetes

The onset of clinical type 1 diabetes is preceded by a long non-symptomatic prodromal period characterized by well-defined stages, which allow the progression towards the symptomatic disease, defined as stage 3, to be predicted [20]. In stage 1, individuals have two or more beta-cell autoantibodies with normal blood sugar, in stage 2, two or more autoantibodies and dysglycemia or glucose intolerance. Given the reduced level of β-cell numbers at the time of diagnosis, the ability to stage type 1 diabetes before clinical onset presents an opportunity to preserve functional residual β-cell mass and prevent the onset of clinical symptoms [21]. The islet-specific autoantibodies are anti-insulin antibodies (IAA), glutamate decarboxylase (GAD), islet antigen 2 (IA-2) and islet-specific zinc transporter (ZnT8). Children with two or more islet autoantibodies in stage 1 have a 5-year risk of clinical T1D of 44%, and a 15-year risk of 80-90%; children with two or more islet autoantibodies in stage 2 have a 5-year risk of clinical T1D of 75% and a lifetime risk of 100% [22]. A child with only one islet autoantibody should be also followed up since could be transient or could develop other autoantibodies and then clinical T1D.

Antibody screening has been used extensively in first-degree relatives of patients with type 1 diabetes (siblings, children, parents), including the TrialNet study, which identified potential subjects for prevention studies and provided information on the natural history of the disease [23]. However, it is known that nearly 90% of children with newly diagnosed T1D have no family history of type 1 diabetes, so simply screening this population misses many cases.

There are many reasons to suggest population screening in Europe [24] and Italy. First, a large number of children have diabetic ketoacidosis at the diagnosis of type 1 diabetes, this number of patients has dramatically increased during the COVID-19 pandemic both in Italy and in the rest of the world [25] [5]. DKA is a serious and life-threatening event associated with short- and long-term sequelae, including significant neurocognitive outcomes, shorter remission phase, lower C-peptide reserve, worse glycaemic control, increased risk of vascular complications, and costs.

Secondly, the screening is cost-effectively. Early detection of T1D in children might possibly reduce the risk or even prevent the deterioration of metabolic function. This would eventually decrease the risk of long-term complications, including brain damage associated with hyperglycaemia and hypoglycaemia, as well as vascular complications. In fact, the analysis of two databases from Sweden, The Swedish Paediatric Diabetes Quality Registry (SWEDIABKIDS) and the Swedish National Diabetes Registry (NDR), found that patients with better metabolic control at the time of stage 3 clinical T1D diagnosis had better metabolic control later in adult life [26]. Other studies have confirmed that lower HbA1c values at diagnosis and early preservation of C-peptide reserve are associated with better metabolic control later in life and reduced risk of long-term complications [27][28]. In addition, early screening for T1D in children could become cost-effective due to cheaper antibody screening methods, prevention of DKA hospitalization and the expected reduction in the incidence and economic impact of diabetes complications [29]. In fact, the Fr1da study aiming to screen 200,000 children aged 3–4 years showed that DKA prevention in about 200 patients may cover a third of the study cost [11]. In Colorado, patients with DKA at diagnosis have had HbA1c 1.4% higher than in those without DKA, for up to 15 years after diagnosis [7]. Moreover, the Autoimmunity Screening for Kids (ASK) has demonstrated that prevention of DKA at diagnosis, combined with persistently lower HbA1c in patients without DKA and reduced incidence of diabetes complications, makes general population screening cost-effective [29].

Thirdly, it should also not forget that early diagnosis of stage 1 or stage 2 T1D could offer children and their families an opportunity to participate in clinical trials, with the aim of delay the clinical manifestations of the disease. There are several trials available in Europe, USA and elsewhere in the world and some drugs have shown promising results in postponing the progression to clinical T1D [30][31][32]. In individuals with a first-degree relative with T1D, one of these drugs (teplizumab, an anti-CD3 monoclonal antibodies) has shown to prolong a diabetes-free time of up to 6 years; this drug has been approved by the Food and Drug Administration (FDA) on November 2022 and it should be soon available in clinical practice.

The advantage of knowing in advance the possibility that a child has T1D and delaying the diagnosis with a drug must be balanced with the anxiety that this information produces in families, and with the efforts and organizational costs that screening requires (Table 1). It has been argued that screening for T1D could induce considerable psychological stress in children diagnosed with pre-symptomatic T1D and their parents (either at risk or in the general population). Natural history studies that have monitored children positive for islet autoantibodies [33][34][35] have reported that parental distress was moderately increased, but returned to baseline levels with an appropriate education and monitoring. Islet autoantibody screening and diagnosis of pre-symptomatic T1D appear unlikely to induce parental psychological stress, which is comparable to that observed in families of children diagnosed with clinical T1D. Data from the Fr1da Study have shown that, when appropriately informed and educated, parents and families of children with two or more autoantibodies had positive feelings toward an early identification of T1D [36].

**Table 1:** Pros and cons of screening for type 1 diabetes

Pros	Cons
Possible prevention of DKA at onset of diabetes	Potential increased anxiety in parents/carers
Opportunity for time to adjust to diagnosis	High numbers of individuals genetically at risk, but who don’t develop T1D
Genetic testing for high-risk genes/genetic risk scores possible at birth for use in combination with autoantibodies	If using IAb alone: - Likely need testing more than once - Will miss those diagnosed before screening and those who seroconvert after screening
IAb detectable with fingerprick test, easy to administer, sensitive and specific	Treatment of early hyperglycaemia can be challenging
May be intervention studies to delay development or prevent T1D	Cost/effectveness still a matter of debate
New drug (teplizumab) available to delay the onset of T1D in high-risk individuals	No drug licensed to definitively prevent diabetes

Modified from Besser R.E.J. et al., Arch Dis Chil 2022

There has been a change towards a more screening-friendly position in recent years, in part because screening for multiple diseases is now possible with broad genetic testing such as exome sequencing. Recently, the European Society of Pediatric Endocrinology (ESPE) approved a Position Statement on Screening for T1D in the general population, hoping that other countries could also support these wishes [37]. The Italian Society of Pediatric Endocrinology and the Italian Society of Pediatrics approved and endorsed this Position Statement, in the meetings of 24 October 2022 and 18 January 2023, respectively. Health authorities would like to see if there is a clear added value, health benefit, and low burden of diagnosing asymptomatic diseases; effective medical care should be available that at least partly prevents or delays symptomatic disease and reduces complications; screening and monitoring need to be cost effective and competitive with respect to other health needs and priorities; and there must be convincing evidence that the false-positive rate is low and identification of false-positive cases does not cause relevant harm. How does screening for beta-cell autoimmunity meet these criteria? Ongoing and future studies will provide information on the prevalence of asymptomatic beta-cell autoimmunity in the general population, program efficacy to prevent DKA and reduce family burden, and precise estimates of the rate of progression to symptomatic disease (i.e., the value predictive positive) or the return to autoantibody negativity (i.e., false positive rate) in children in the general population. Experiences from European countries and the United States, data on the added value of improved long-term care and the reduction of complications, and long-term data on the burden and social implications for families are essential. We will soon have a treatment that delays the disease (teplizumab) available in clinical practice, however the economic benefit of such a treatment is still unknown. If the authorities and insurers accept the screening and treatment of children at risk, organizational efforts and urgent investments will be required [24] [38]. In any case, the voice of patients and their families must be recognized and considered in the decision-making process.

## Conclusion

In summary, screening for asymptomatic β-cell autoimmunity is possible, is effective in prevention of DKA in children and should be implemented. We do believe that European and Italian authorities should endorse this need in order to early detect children at risk when the disease is in stage 1 or stage 2. This could possibly preserve the beta-cell mass, allow a longer insulin independence and prevent short-term and long-term complications.

## Electronic supplementary material

Below is the link to the electronic supplementary material.

Supplementary Material 1: Point-by-point response

## Data Availability

All studies and data analyzed during this study are included in this article. Further enquiries can be directed to the corresponding author.
