# Supplementary material for: Autoantibody test for type 1 diabetes in children: are there reasons to implement a screening program in the general population? A statement endorsed by the Italian Society for Paediatric Endocrinology and Diabetes (SIEDP-ISPED) and the Italian Society of Paediatrics (SIP)
Source: Ital J Pediatr. 2023 Jul 19;49:87. doi: 10.1186/s13052-023-01438-3 (PMC10354886; doi:10.1186/s13052-023-01438-3)
Supplement: Supplementary file 1 — Supplementary Material 1: Point-by-point response [file 13052_2023_1438_MOESM1_ESM.docx]

**Point-by-point response**

Reviewer reports:

**Reviewer #1: Valentino Cherubini and Francesco Chiarelli present a statement of the SIEDP-ISPED Society endorsing the screening in the general population for autoantibody testing for T1D in children. A similar article was published in June in Horm Res Pediatr (Chiarelli F, et al. Screening of Islet Autoantibodies for Children in the General Population: A Position Statement Endorsed by the European Society for Paediatric Endocrinology. Horm Res Paediatr. 2022;95(4):393-396) dealing with the same issue but on behalf of ESPE.
Despite the authors are expert in the field and the manuscript is formally well written, I have the following concerns.
1-Since the authors claims that the endorsement toward this type of screening is presented on behalf of the SIEDP-ISPED and do not represent only the personal opinion of the two authors, it is at least surprising that neither the president of the Society, nor the council are co-Author of the manuscript. They are just mentioned in the Acknowledgements. I am sure that the preliminary endorsement of such a screening would be the matter of a deep debate within the Society. Therefore, I would eliminate from the title 'A Statement of the Italian Society for Paediatric Endocrinology and Diabetes (SIEDP-ISPED)'. By contrast, if the authors do not want a eliminate the role of the SIEDP-ISPED, it should be clearly stated where and when the controversial issue was discussed within the society.**

Thanks for this comment. The ISPED Council discussed and approved the ESPE Position Statement on Screening of Islet Autoantibodies for Children in the General Population in the Council Meeting on 24 October 2022 (minutes attached). The Italian Society of Pediatrics has also endorsed the document in the Council Meeting on 18 January 2023 (minutes attached) (The minutes are in Italian).

Following your suggestion, we have updated the text as follows:

Recently, the European Society of Pediatric Endocrinology (ESPE) approved a Position Statement on Screening for T1D in the general population, hoping that other countries could also support these wishes (37). The Italian Society of Pediatrics and the Italian Society of Pediatric Endocrinology approved this Position Statement, respectively in the meetings of 18 January 2023 and 24 October 2022.

**2- My suggestion is that the authors simply present pros and cons on the problem, by enlarging the issues presented in table 1. As an example, it is well known that T1D children are currently treated much better than in the past and that life expectancy has consistently improved in recent years, thanks to the improved metabolic control. A number of editorials and reviews have been published underlining how quality of life has recently improved using technologies. Despite the authors well describe the burden of diabetes management, both aspects should be described, otherwise the 'statement' may not be objective**.

Thank you for this point.

We updated the text as follows:

Numerous studies have shown that new technologies improve glycemic control and long-term outcomes in children and adolescents with type 1 diabetes. Furthermore, these devices have improved quality of life and have patient satisfaction (Health-related quality of life and treatment preferences in adolescents with type 1 diabetes. The VIPKIDS study. Cherubini V, Gesuita R, Bonfanti R, Franzese A, Frongia AP, Iafusco D, Iannilli A, Lombardo F, Rabbone I, Sabbion A, Salvatoni A, Scaramuzza A, Schiaffini R, Sulli N, Toni S, Tumini S, Mosca A, Carle F; VIPKIDS Study Group. Acta Diabetol. 2014 Feb;51(1):43-51. doi: 10.1007/s00592-013-0466-x. – Reference #4).

**3- ISPAD guidelines (chapter 3.3.2) report 'Given the current inability to intervene effectively in the T1D disease process, international debate continues about whether screening should be population-wide or limited to first-degree family members.' Therefore, I think it is premature to state on behalf of a scientific society that the population screening for pre-symptomatic T1D is certainly convenient.**

Thank you for this comment.

The ISPAD 2022 guidelines, in which Valentino Cherubini collaborated, Chapter 11: DIABETIC KETOACIDOSIS AND HYPEROSMOLAR STATE, underline the importance of prevention of DKA. On the other hand, in children at risk of diabetes identified through islet cell antibody screening programs there is a dramatic reduction in the frequency of DKA at the clinical onset of the disease.

Furthermore, in Chapter 3.0 of the ISPAD guidelines, "EXECUTIVE SUMMARY: RECOMMENDATIONS AND PRINCIPLES", it is reported that

General population screening programs using combinations of genetic and autoantibody testing can identify high-risk children (evidence A).

Nevertheless, we do not state that "on behalf of a scientific society that the population screening for pre-symptomatic T1D is certainly convenient". On the other hand, we suggested that "screening for asymptomatic β-cell autoimmunity is possible, effective in prevention of DKA in children and should be implemented".

**4- One of the authors declares a relationship with Provention Bio: is not it a conflict of interest?**

Thanks for this comment.

Author Francesco Chiarelli, who has a scientific collaborative relationship with Provention Bio, only potentially has a conflict of interest related to this article. In fact, the document discusses the possibility of implementing a screening program for diabetes and not treatment with Teplizumab (manufactured by Provention Bio), which the Food and Drug Administration recently (November 17^th^ 2022) approved to delay the onset of type 1 diabetes in children older than 8 years. Moreover, it should be remembered that the drug is not yet authorized by the EMA. However, even though this is only a potential conflict, the author did state this condition, as is usually the case in the scientific literature.


**Reviewer #2: In this manuscript, the authors aimed to discuss the main unmet needs in type 1 diabetes and the opportunity presented by population screening. They declared that the Italian Society for Paediatric Endocrinology and Diabetes (SIEDP/ISPED) endorses this document with the specific aim of increasing awareness concerning screening for pediatric T1D in the general population.
They underlined that the drugs and technologies have determined improvements in the care of patients with type 1 diabetes; however, some major unmet needs remain unresolved, including the high frequency of diabetic ketoacidosis (DKA), the continued threat of hypoglycemia, the day-to-day burden of diabetes management, and failure to achieve optimal glycemic control.**
**After having over-acclaimed the unmet needs I feel that they overindulged in highlighting that screening for asymptomatic β-cell autoimmunity is advisable and overall effective in preventing DKA, without real data and should be implemented.  They stated that the Italian Society for Pediatric Endocrinology and Diabetology (ISPED) has endorsed a Position Statement on Screening for T1D in the general population but without citing any reference.**

Thanks for this suggestions.

We have partially modified the text and updated the text as follows:

Recently, the European Society of Pediatric Endocrinology (ESPE) approved a Position Statement on Screening for T1D in the general population, hoping that other countries could also support these wishes (37). The Italian Society of Pediatrics and the Italian Society of Pediatric Endocrinology approved this Position Statement, respectively in the meetings of 18 January 2023 and 24 October 2022.

**I think that this manuscript even if interesting and well-written reflects only the thought of the two authors and as interesting as it may be it cannot be defined as a SIEDP/ISPED statement. This is not a Society Consensus also because ISPED Council members are cited only in the Acknowledgement session and not enrolled in the authorship.
Therefore I suggest removing from the title "A Statement of the Italian Society for Paediatric Endocrinology and Diabetes (SIEDP-ISPED)" and writing in the conclusion that the issue discussed in this manuscript could be the object of a future SIEDP/ISPED Consensus.**

The Italian Society of Pediatrics and the Italian Society for Pediatric and Adolescent Endocrinology have just ‘endorsed’ the document respectively in the meetings of 18 January 2023 and 24 October 2022 (we have provided the minutes of the two societies, in Italian).

**Furthermore, I suggest to mitigate the underlining of unmet needs of type 1 diabetes, also because this patient population, who can no longer prevent its pathology, has to go on living serenely with its disease.**

We have followed the suggestions. Thanks.


**Reviewer #3: The manuscript is original and well written.  The topic is highly innovative and provides new information an possible future scenarios**

We thanks this reviewer for the support.


**Reviewer #4: Review of the manuscript ITJP-D-22-00648 by Cherubini V and Chiarelli F : this is really an interesting paper dealing with an important problem in pediatric diabetology.
The paper is well written and the problem of the screening in the general population is really an "hot" topic in this period of time, moreover after the statement of the FDA in novembre 2022.
Probabilly the Teplizumab will be approved as well in Europe by EMA in the near future, and all pediatric diabetology will face the question of screening of patients and treatment of subjects positive for antibodies tests.
However, i suggest to modifye the title,  because this is not a statement of SIEDP, but the SIEDP endorsed a statement by ESPE, as is stated correctly in the paper.
I suggest to publish this paper.**

We have modified the paper and the statements, underlining that the Italian Society of Pediatrics and the Italian Society for Pediatric and Adolescent Endocrinology have just ‘endorsed’ the document respectively in the meetings of 18 January 2023 and 24 October 2022 (we have provided the minutes of the two societies, in Italian).
